# Tissue-Specific Transcriptome and Hormonal Regulation of Pollinated and Parthenocarpic Fig (*Ficus carica* L.) Fruit Suggest that Fruit Ripening Is Coordinated by the Reproductive Part of the Syconium

**DOI:** 10.3389/fpls.2016.01696

**Published:** 2016-11-29

**Authors:** Yogev Rosianski, Adi Doron-Faigenboim, Zohar E. Freiman, Kumar Lama, Shira Milo-Cochavi, Yardena Dahan, Zohar Kerem, Moshe A. Flaishman

**Affiliations:** ^1^Institute of Plant Sciences, The Volcani Center, Agricultural Research OrganizationBet-Dagan, Israel; ^2^The Robert H. Smith Faculty of Agriculture, Food and Environment, The Hebrew University of JerusalemRehovot, Israel

**Keywords:** *Ficus carica*, pollinated vs. parthenocarpic fruit, tissue-specific transcriptome, fruit ripening, abscisic acid, ethylene

## Abstract

In the unconventional climacteric fig (*Ficus carica*) fruit, pollinated and parthenocarpic fruit of the same genotype exhibit different ripening characteristics. Integrative comparative analyses of tissue-specific transcript and of hormone levels during fruit repining from pollinated vs. parthenocarpic fig fruit were employed to unravel the similarities and differences in their regulatory processes during fruit repining. Assembling tissue-specific transcripts into 147,000 transcripts with 53,000 annotated genes provided new insights into the spatial distribution of many classes of regulatory and structural genes, including those related to color, taste and aroma, storage, protein degradation, seeds and embryos, chlorophyll, and hormones. Comparison of the pollinated and parthenocarpic tissues during fruit ripening showed differential gene expression, especially in the fruit inflorescence. The distinct physiological green phase II and ripening phase III differed significantly in their gene-transcript patterns in both pulp and inflorescence tissues. Gas chromatographic analysis of whole fruits enabled the first determination of ripening-related hormone levels from pollinated and non-pollinated figs. Ethylene and auxin both increased during fruit ripening, irrespective of pollination, whereas no production of active gibberellins or cytokinins was found in parthenocarpic or pollinated ripening fruit. Tissue-specific transcriptome revealed apparent different metabolic gene patterns for ethylene, auxin and ABA in pollinated vs. parthenocarpic fruit, mostly in the fruit inflorescence. Our results demonstrate that the production of abscisic acid (ABA), non-active ABA–GE conjugate and non-active indoleacetic acid (IAA)–Asp conjugate in pollinated fruits is much higher than in parthenocarpic fruits. We suggest that fruit ripening is coordinated by the reproductive part of the syconium and the differences in ABA production between pollinated and parthenocarpic fig fruit might be the key to their different ripening characteristics.

## Introduction

Plant hormones have long been documented for their key role in synchronizing signals between the developing seed and fruit tissue. The onset of the ripening process, when seeds reach maturity, is considered a hormonal turning point, during which hormone levels within the fruit undergo major alterations involving an overall decrease in auxin, gibberellin, and cytokinin, and a simultaneous increase in abscisic acid (ABA) and ethylene (Crane, 1964, 1969; Srivastava and Handa, 2005; McAtee et al., 2013). Reduction in auxin and cytokinin is the key to initiating the ripening process of fruit maturation. Genetic studies of tomato *ripening inhibitor* (*rin*) mutant and suppression of a *rin*-like *MADS*-box gene in apple showed a high auxin ratio in fruit that did not ripen (Rolle and Chism, 1989; Schaffer et al., 2013; Ireland et al., 2013). Decreases in free cytokinin and auxin levels were also observed before ripening in orange and grape (Minana et al., 1989; Bottcher et al., 2010).

Ethylene is considered to be the ‘ripening’ hormone in climacteric fruit. Ethylene biosynthesis is initiated by the conversion of methionine to *S*-adenosylmethionine (SAM) via SAM synthase. SAM is converted to amino-cyclopropane carboxylate (ACC) by ACC synthase, and then transformed to ethylene by ACC oxidase (ACO) (Bleecker and Kende, 2000; Wang et al., 2002). Ethylene content increases at ripening onset in climacteric fruit, such as tomato, apple and mango (Giovannoni, 2001; Adams-Phillips et al., 2004; Seymour et al., 2012; Zaharah et al., 2012). In contrast, no such burst in ethylene levels can be found in non-climacteric fruit such as strawberry, citrus, and grape (Seymour et al., 2012; Symons et al., 2012). Climacteric ethylene burst has also been documented in fig (*Ficus carica* L.) fruit. However, a molecular study of ethylene-related genes by Freiman et al. (2015) revealed additional non-climacteric autoinhibition of ethylene production, as previously documented by Marei and Crane (1971).

Production of the hormone ABA, involved in many plant processes such as seed development, dormancy, fruit development and plant response to environmental stresses (Zeevaart and Creelman, 1988; Xiong and Zhu, 2003; Setha, 2012; Leng et al., 2014), has been shown to precede ethylene production in climacteric fruits (Lara and Vendrell, 2000; Symons et al., 2012; Leng et al., 2014). ABA accumulation is high at the onset of or during ripening of climacteric fruit (Leng et al., 2014). Inadequate ABA production affects fruit growth and delays chlorophyll degradation (Rodrigo et al., 2003; Galpaz et al., 2008). ABA-biosynthesis pathways have long been studied; zeaxanthin epoxidase (ZEP), 9-cis-epoxycarotenoid dioxygenase (NCED), short-chain alcohol dehydrogenase (ABA2) and abscisic aldehyde oxidase (AAO) have all been found to regulate ABA synthesis (Schwartz, 2003; Taylor et al., 2005). Moreover, downregulation of ABA content occurs via degradation to either phaseic acid (PA) or dihydrophaseic acid (DPA) initiated by the action of ABA-8′-hydroxylase (ABA-8′-h) or via reversible conjugation to glucose by ABA–glucose ester conjugate (ABA–GE) (Setha et al., 2005; Jiang and Hartung, 2008; Seo and Koshiba, 2011).

Parthenocarpy is believed to be triggered by specific plant hormones. Studies of several fruit crops have revealed that endogenous hormone levels of parthenocarpic cultivars are much higher than those of non-parthenocarpic ones at early stages of fruit development (Mapelli et al., 1978). In tomato, external application of auxin or gibberellic acid (GA) induces parthenocarpic fruit (Gustafson, 1937; Wittwer et al., 1957). The parthenocarpic *pat* tomato mutant showed threefold higher endogenous levels of auxin-like substances than the normal plant, whereas GA levels were higher during the early fruit growth period and cytokinin levels were lower throughout fruit growth than in normal pollinated fruit (Mapelli, 1981). The changes in auxin and GA levels in pollination-dependent and parthenocarpic tomato fruit set were recently characterized using RNA sequencing (RNA-Seq) of the transcriptomes (Tang et al., 2015). In contrast to endogenous hormones in non-pollinated ovaries seem to decrease prior to ripening and fruit senescence (Pharis and King, 1985; Gillaspy et al., 1993).

The fig fruit bears a unique closed inflorescence structure—the syconium—which is a multiple fruit composed of small individual seeds and drupelets that develop from the ovaries enclosed in the succulent receptacle to form a single accessory fruit (Storey, 1977; Flaishman et al., 2008). Development of the fig’s female fruit is characterized by a double sigmoid growth curve comprised of three phases (Marei and Crane, 1971). Phase I is characterized by a rapid growth in size. During phase II, the fruit remains nearly the same size, color, and firmness. Phase III is considered to be the ripening phase, during which the fruit grows, its color changes, and the pulp texture softens and changes to an edible state. In the parthenocarpic fruit of the purple female fig cv. Brown Turkey, ethylene production rises when the green hue of the peel starts to fade to a yellowish shade, at the transition from developmental phase II to III. Early fig fruit studies showed three distinct peaks in auxin production: one at the end of first fruit growth in phase I, another at the end of phase II and a third during the rapid fruit growth in phase III (Lodhi et al., 1969). Parthenocarpy in ‘Calimyrna’ fig fruit was successfully induced by the application of cytokinin, auxin and GA but not transferred into practice. These three types of endogenous hormones are thought to originate in the seeds and to stimulate fruit growth directly (Crane and Blondeau, 1949; Crane, 1965; Crane and van Overbeek, 1965).

Recently, a comparative physiological and morphological analysis of pollination effects on the ‘common fig’-type ‘Brown Turkey’ fruit development and ripening characteristics was performed (Rosianski et al., 2016). In general, pollinated fruit showed altered developmental processes compared to parthenocarpic fruit. Ripe pollinated fruit were round, in contrast to the pear-like shape of the parthenocarpic fruit, and they were larger in both diameter and weight, with improved firmness compared to the parthenocarpic fruit. At harvest, pollinated fruit exhibited commercially desirable physical and taste characteristics with advanced fertile nutlets compared to the sterile undeveloped non-bearing nutlets of the parthenocarpic fruit (Rosianski et al., 2016).

In this study, a comprehensive high-throughput Illumina RNA-Seq transcriptome of parthenocarpic and pollinated fig pulp and inflorescence tissues at five developmental stages was assembled. Integrative comparative analyses of tissue-specific transcript and hormone levels in both pollinated and parthenocarpic fig fruit were employed to unravel the similarities and differences in regulatory processes during fig fruit ripening. In this study we present the hormonal regulation of the unusual climacteric fig fruit type during the ripening. We used the pollinated versus parthenocarpic fruits of the same genotype to emphasize these processes.

## Materials and Methods

### Plant Material and Treatment

Mature fig trees (*Ficus carica* L., cv. Brown Turkey) grown in a commercial orchard near Beer Tuvia, Israel (31°44′13.66″N, 34°43′32.05″E) were used for two field experiments conducted in May 2012 and 2013. The Brown Turkey cultivar belongs to the ‘common fig’ type that produces parthenocarpic fruit. All trees were maintained according to commercial-production practices with short pruning trimming up to biennial branches such that the braking buds are 2 years old.

**FIGURE 1:**
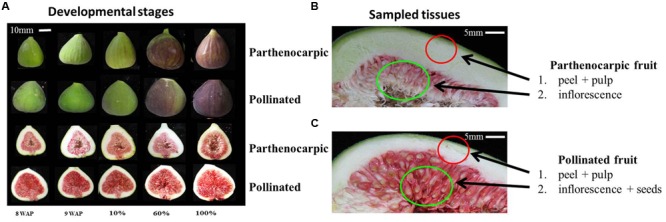
**Fig fruit developmental stages and tissues used to extract RNA and construct cDNA libraries for the Illumina high-throughput sequencing. (A)** Developmental stages of parthenocarpic (top rows of each set) and pollinated (lower rows of each set) fruit displaying pulp and inflorescence; 8WAP – 8 weeks after pollination; 9WAP – 9 weeks after pollination; 10% – 10% fruit ripening; 60% – 60% fruit ripening; 100% – 100% fruit ripening. **(B)** Parthenocarpic sampled tissues that were used in this study. (**C**) Pollinated sampled tissues that were used in this study.

Fruit corresponding to growth phase I (15 mm in diameter, *n* = 2,000), positioned sixth or seventh on the shoot, were tagged on the trees; 1,000 fruit, covered with 100-mesh bags to prevent natural pollination by wasps, served as the non-pollinated control population, while the other 1,000 were hand-pollinated as follows: several ‘caprifig’-type fig fruits of selected varieties were harvested at the ripening phase. Fruits were cross-sectioned to reveal the stamens. The back of every half was gently tapped to shake the pollen into a small vial which was immediately stored at -20°C. Pollen (0.5 g) was dissolved in 100 mL 2% sucrose solution (2 g sucrose in 100 mL ddH_2_O) and injected into the fruit through the ostiole with a plastic syringe. To ensure that the entire inflorescence comes into contact with the pollination solution, fruit were filled until a drop of the pollination solution came out through the ostiole. The pollination solution volume injected into each individual fruit thus varied with the size of the inner fruit cavity. Following hand-pollination, all fruits were covered with 100-mesh bags to prevent natural pollination. Fruits from each treatment were collected every 7 days for 90 days post-pollination to produce a developmental profile. At 90 days after pollination, all ripened fruit were harvested and classified according to their ripening stage, which was determined by the percentage of the red color coverage of the outer fruit skin (**Figure 1**).

For the high-throughput sequencing, three biological replicates were collected from pools of four fruit at each developmental stage that were dissected into pulp and inflorescence and immediately frozen in liquid N_2_, pulverized and stored at -80°C. For the hormone analysis, 15 whole fruits from each developmental stage were pooled and immediately frozen in liquid N_2_, pulverized, lyophilized and stored at -20°C.

### Hormonal Content Analysis

About 50 mg of two lyophilized biological replicates from each sample were prepared for hormone analysis. Quantification of ABA, ABA metabolites, indoleacetic acid (IAA), IAA metabolites, gibberellins, gibberellin metabolites and cytokinins was conducted at the National Research Council of Canada (Saskatoon, SK, Canada) according to their published protocols^1^.

### RNA Extraction

Total RNA was isolated from pulp (peel and receptacle) and inflorescence according to Jaakola et al. (2001). RNA concentration was determined in a NanoDrop ND-1000 spectrophotometer and its integrity was checked by running 1 μL in a 1% (w/v) agarose gel stained with Bromophenol blue.

### Paired-End mRNA-Seq Library Preparation and Sequence Generation

Total RNA was extracted from 40 tissue samples. RNA fragmentation, double-stranded cDNA synthesis and adaptor ligation were performed using the Truseq^TM^ RNA Sample Prep Kit-v2 (Illumina, San Diego, CA, USA) according to the manufacturer’s instructions. Library concentration and size were assayed using the Qubit^®^ dsDNA HS Assay Kit in a Qubit^®^ 2.0 Fluorometer (Invitrogen, Lidingo, Sweden) and the Agilent DNA1000 Kit on a 2100 tape station (Agilent Technologies, Nærum, Denmark). Each library was normalized to a final concentration of 100 nM. All 22 samples were grouped into pools and diluted to a final concentration of 10 nM. Libraries were sequenced on a genome analyzer equipped with a paired-end module (Illumina) at the Technion – Israel Institute of Technology (Haifa, Israel) to generate 100-bp paired-end reads.

### *De novo* Transcriptome Assembly

Raw reads were subjected to a filtering and cleaning procedure using the FASTX Toolkit ^2^ (version 0.0.13.2) as follows: (i) read-end nucleotides with quality scores <30 were trimmed using the fastq_quality_trimmer; (ii) reads with less than 70% base pairs with quality score ≤30 were removed using the fastq_quality_filter. A total of ∼1.9 billion cleaned reads, obtained after processing and cleaning, were assembled de novo using Trinity software [version trinityrnaseq_ r20131110 (Grabherr et al., 2011); 25 mer k-mer size]. Filtering of the likely contig artifacts was carried out as follows: (i) abundance estimates were calculated for each contig using the RSEM software; (ii) only contigs representing more than 1% of the per-component (IsoPct) expression level were retained. The resulting de-novo assembly-generated transcriptome catalog is shown in **Table 1**.

**Table 1:** Statistical summary of parthenocarpic and pollinated ripening fig fruit transcriptome catalogs.

	Total trinity	Contig	Median contig	Average	Total assembled			
	Transcripts	N50	Length	Contig	Bases	Annotated NCBI	GOs	Malus
Full assembly	147051	1728	459	903.71	1.30E+08	53938	36474	49128

### Data Availability

The transcriptome datasets are available in the NCBI Sequence Read Archive (SRA) under BioProject accession PRJNA322124.

### Sequence Similarity and Functional Annotation

The resulting contigs were annotated using the Basic Local Alignment Search Tool (BLASTX) (Altschul et al., 1990) against the NCBI non-redundant (Nr) and *Malus* × *domestica* protein (from the genome database for Rosaceae) that chosen for being a fleshy climacteric fruit like the *Ficus carica* and for being well annotated in the NCBI, databases with an *E*-value cut-off of 10^-5^ and TAIR. Blast2GO software (Conesa et al., 2005) was used for gene ontology (GO) classification^3^.

### Differential Expression and Cluster Analysis

Transcript quantification (number of reads per gene) from the RNA-Seq data was performed using the bowtie aligner (Langmead et al., 2009) and the expectation-maximization method (RSEM), which handles read-mapping uncertainty with a statistical model by estimating maximum-likelihood expression levels (Li and Dewey, 2011). Differential expression analysis was performed with the edgeR package (Robinson et al., 2010). Transcripts with more than twofold differential expression levels with false discovery-corrected statistical significance of at most 0.001 were considered differentially expressed (Benjamini and Hochberg, 1995). The expression patterns of the transcripts in the different samples were studied using cluster analysis of the differentially expressed transcripts in at least one pairwise sample comparison. Then, the Trinity protocol (Haas et al., 2013) expression normalization was designed using trimmed mean of M-values (TMM), following fragments per feature kilobase per million reads mapped (FPKM) calculations. Hierarchical clustering of gene expression and visualization of heat maps were performed using R Bioconductor (Gentleman et al., 2004). We used the tool at http://bioinformatics.psb.ugent.be/webtools/Venn/ for Venn diagram construction.

### GO-Enrichment Analysis

Gene ontology-enrichment analysis was carried out using the Blast2GO (Conesa et al., 2005) program based on Fisher’s Exact Test (Upton, 1992) with multiple testing correction of false discovery rate (FDR) (Benjamini and Hochberg, 1995). The threshold was set as a FDR with corrected *P*-value of less than 0.05. GO analysis was performed by comparing the GO terms in the test sample to those in a background reference. The REVIGO web server (Supek et al., 2011) was used for reduction and visualization of the GO terms. A hypergeometric test using a MATLAB script was performed for the detection of significantly enriched pathways in a gene list with a cut-off *P*-value < 0.05.

## Results

### Generation, *De novo* Assembly and Annotation of Parthenocarpic and Pollinated Ripening Fig Transcriptome

To establish the transcriptome of ripening parthenocarpic and pollinated fig fruit, 40 cDNA libraries from fig fruit pulp and inflorescence at five developmental stages: (i) 8 weeks after pollination (8WAP), (ii) 9WAP, both from fruit developmental phase II, (iii) 10% fruit ripening, where 10% of the fruit surface turned to red (iv) 60% fruit ripening, where 60% of the fruit surface turned to red and (v) 100% fruit ripening, from fruit developmental phase III, were created (**Figure 1**). Illumina HiSeq 2000 was used for sequencing, yielding 803.4 million 100-bp paired-end reads with an average of 20 million reads per sample. Properties of the *de novo* assembly after quality trimming and filtration are shown in **Table 1**. For gene annotation, the assembled transcript datasets were compared with the NCBI and *Malus* × *domestica* protein databases using the BLASTX program (Altschul et al., 1990), resulting in 53,938 (36.7%) and 49,128 (33.4%) annotated genes for parthenocarpic and pollinated fig fruit, respectively. GO classification via BLAST2GO revealed high representation of cellular metabolic processes, primary metabolic processes, organic substance metabolic processes, biosynthetic processes and macromolecular metabolic processes in the parental category. In addition a high representation of binding (including ion, protein, heterocyclic and organic cyclic compound binding), hydrolase, transferase and oxidoreductase activities in the molecular function parental category, and high representation of cell, intracellular, cytoplasm, organelle and membrane-bound organelle in the cellular component category, were confirmed (**Supplementary Figure S1**).

### Gene-Expression Pattern during Fruit Development

**FIGURE 2:**
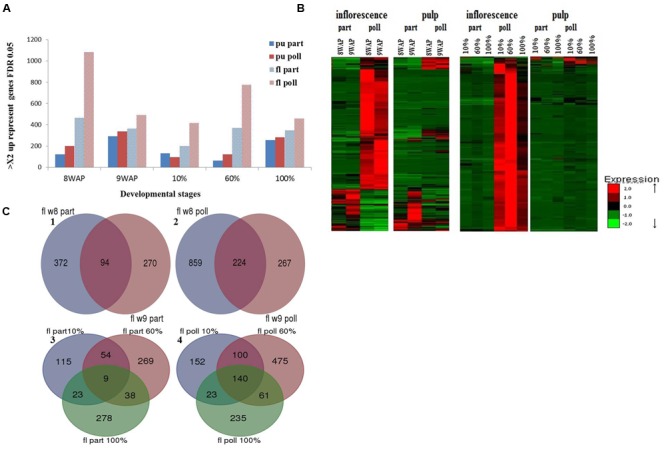
**Differentially expressed genes in fruit pulp vs. inflorescence at the five developmental stages. (A)** Number of parthenocarpic- (part; blue) and pollination-induced (poll; red) genes, FDR < 0.001, >2 fold higher expression (FPKM) levels. **(B)** Heat map of the upregulated genes in parthenocarpic and pollinated pulp and inflorescence. **(C)** Venn diagram of upregulated genes in the inflorescence during: (1) parthenocarpic phase II, (2) pollinated phase II, (3) parthenocarpic phase III, (4) pollinated phase III.

To evaluate gene expression during fruit development for each of the four different treated tissues: (i) parthenocarpic inflorescence, (ii) pollinated inflorescence, (iii) parthenocarpic pulp and (iv) pollinated pulp, differentially expressed transcripts were evaluated using 10 pair wise comparisons (all vs. all, edgeR, FDR < 0.001, >2 fold, **Figure 2A**). Hierarchical clustering analysis of gene representations in parthenocarpic and pollinated inflorescence and pulp revealed four distinct cluster patterns for each of the four different tissues (**Supplementary Figure S2**). The gene-representation expression patterns in the different hierarchical clusters clearly distinguished between developmental phases II and III and could be classified into two major groups: genes whose expression declines when entering ripening phase III and genes that have increased levels at this developmental stage (**Supplementary Figure S2**). The distinct gene-expression behavior between phases II and III allowed us to look further at the genes that are activated in each phase (phase II – 8WAP and 9WAP, and phase III – 10, 60, and 100% ripening) as one group for each phase.

For each hierarchical cluster, GO-enrichment analysis was performed to shed light on the most prominent molecular functions and biological processes during ripening of the specific parthenocarpic or pollinated tissue. While the number of downregulated and upregulated enriched molecular functions was similar in the pollinated inflorescence, the parthenocarpic inflorescence showed a similar number of downregulated genes but three times higher enrichment in upregulated genes. The major upregulated enriched genes in the parthenocarpic inflorescence were involved in the cellular response to internal and external signals and protein metabolism (**Supplementary Table S1**). While the number of upregulated genes in the parthenocarpic pulp was similar to that in the parthenocarpic inflorescence, the pollinated pulp showed almost three times higher enrichment compare to the pollinated inflorescence (**Supplementary Table S1**). In addition to the cellular responses and protein metabolism, the pollinated pulp showed upregulation and enrichment in hormone-related gene expression (**Supplementary Table S1**).

### Genes that are Differentially Expressed in Parthenocarpic vs. Pollinated Fruits

To evaluate the effect of pollination on fig fruit development and ripening, a set of differentially expressed genes in either pulp or inflorescence at each developmental stage was mined. All of these genes showed a minimum twofold higher FPKM with FDR threshold of 0.05 (edgeR) (**Figure 2**).

In general, the number of differentially expressed genes in pollinated vs. parthenocarpic inflorescences was higher than the number in pollinated vs. parthenocarpic pulp (**Figure 2**). Except for the 10% ripened pulp sample, the number of upregulated genes in all pollinated samples was higher than in their parthenocarpic counterparts (**Figure 2**). Hierarchical cluster analysis of gene-expression patterns revealed four significantly different groups of up-represented genes during phase II (parthenocarpic inflorescence and pulp, pollinated inflorescence and pulp) and two groups in phase III: a major group of up-represented genes in the pollinated inflorescences and a small group in the pollinated pulp (**Figure 2B**). To calculate the intersections of molecular function lists that share higher representation in all parthenocarpic or pollinated phase II or III stages, we used the public web tool http://bioinformatics.psb.ugent.be/webtools/Venn/ to create a Venn diagram and list for each group (**Figure 2C, Table 2**). In general, the number of up-represented genes throughout phases II and III in the inflorescence was higher than in the pulp, while a larger number of up-represented genes was found in phase II compared to phase III (**Table 2**). The number of up-represented genes in the pollinated phase II inflorescence was ca. double that in the pollinated phase III inflorescence and three times that in the parthenocarpic phase II inflorescence (**Table 2**). The number of up-represented genes in pollinated phase II pulp was four times lower than that in the pollinated phase II inflorescence but ca. three times higher than that in parthenocarpic phase II pulp (**Table 2**). The up-represented genes in the pollinated phase III inflorescence were the only substantial up-representation in any of the phase III samples (**Table 2**). The molecular functions color, taste and aroma, storage, seeds and embryo, chlorophyll, and hormone-related function genes had a relatively high number of up-represented genes in the pollinated inflorescence over the parthenocarpic inflorescence during developmental phases II and III (**Table 2**). No other significantly up-represented genes in one tissue or treatment over the other could be seen in phase III. In phase II, the parthenocarpic inflorescence showed up-representation of protein degradation and cell response genes relative to the pollinated inflorescence. In the phase II pulp tissue, the pollinated fruits showed up-representation of storage, protein degradation and cell response-related genes relative to the parthenocarpic pulp, while the parthenocarpic pulp showed up-representation of cell response-related genes relative to the pollinated pulp (**Table 2**).

**Table 2:** Up-represented molecular function groups for each development phase in parthenocarpic (part) and pollinated (poll) inflorescence and pulp tissues.

	phase II				phase III			
	Inflorescence part up	Inflorescence poll up	Pulp part up	Pulp poll up	Inflorescence part up	Inflorescence poll up	Pulp part up	Pulp poll up
Unknown	16	29	2	5	3	17	–	–
Color taste and aroma	2	15	3	1	–	13	–	–
Storage	7	23	1	8	3	16	–	1
Protein degradation	20	34	1	7	–	18	–	3
Seeds and embrvo	1	13	–	2	–	15	–	–
Chlorophyll	3	10	–	3	–	11	–	–
Cell response	22	37	8	15	–	17	–	–
Hormones	3	14	3	4	–	4	–	–
TF and response element	11	33	3	6	1	17	–	1
Cell energy	4	10	1	3	2	6	–	–
Cell wall	5	6	–	4	–	6	–	–
Total	94	224	22	58	9	140	–	5

### Hormone Production during the Parthenocarpic and Pollinated Fig Fruit Ripening Process

**FIGURE 3:**
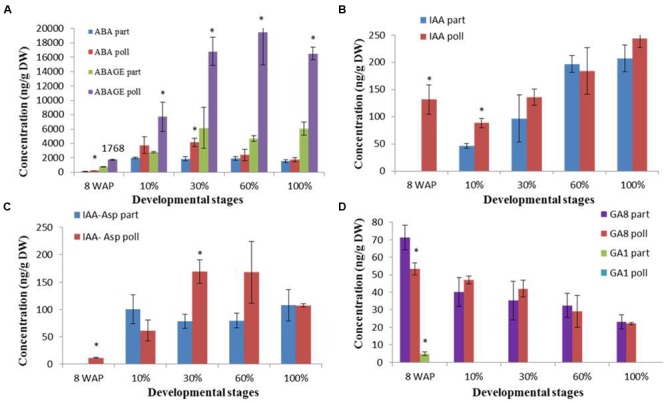
**Hormone concentrations during parthenocarpic (part) and pollinated (poll) fig fruit development and ripening. (A)** ABA and ABA–GE (ABAGE). **(B)** IAA. **(C)** The IAA–Asp conjugate. **(D)** GA1 and GA8. Average ± SE of two replicates of 15 fruits per treatment. Bars represent standard error. Asterisk represent statistical significance determined using Student’s *t*-test.

In this study, for the first time, a ripening-related hormotome revealed the levels of ABA, IAA, GA, cytokinins and their derivatives during the ripening of parthenocarpic and pollinated fig fruit (**Figure 3, Supplementary Figure S3**). Whole-fruit ABA content increased during fruit ripening with a maximum of 4,170 ng/g DW in pollinated 30% ripened fruit compared to a maximum of 2,016 ng/g DW in parthenocarpic 10% ripened fruit, and declined toward the 100% ripe stage with ABA contents of 1,743 and 1,577 ng/g DW in pollinated and parthenocarpic fruit, respectively (**Figure 3A**). The prominent ABA-degradation pathway seemed to be via the ABA–GE conjugate as its concentration was three to four times higher in both parthenocarpic and pollinated fruit than the concentration of ABA, showing a maximum of 6,182 ng/g DW in the 30% ripened parthenocarpic fruit and 19,507 ng/g DW in the pollinated 60% ripened fruit, while no decline in ABA–GE was detected in either case (**Figure 3A**). The second ABA-degradation pathway that goes through PA toward DPA was one order of magnitude lower than the ABA content. While the PA content showed no differences between parthenocarpic and pollinated fruit, with a maximum 50 and 125 ng/g DW in the 30% ripened fruit, respectively, the DPA content in the pollinated fruit reached a maximum 221 ng/g DW at the 30% ripening stage, two times higher than the maximum content of DPA, 137 ng/g DW, in parthenocarpic fruit at the 60% ripening point (**Supplementary Figure S3A**). Endogenous auxin, which was represented by the biologically active IAA and its non-active conjugate with aspartic acid IAA–Asp was detected in all examined samples (**Figures 3B,C**). While IAA content increased throughout the entire ripening period, showing no significant differences between parthenocarpic and pollinated fruit, the IAA–Asp conjugate maintained constant levels in all 10–100% ripened parthenocarpic fruit with peaks at the 30 and 60% ripening stages of 168 ng/g DW (**Figure 3C**).

The only active GA during the ripening process was GA8, which is the GA1 inactivator. GA8 content showed no differences between parthenocarpic and pollinated fruit throughout the entire ripening process, displaying some decline from 53 ng/g DW and 71 ng/g DW 8WAP to 23 ng/g DW and 23 ng/g DW at the 100% ripening stage, respectively (**Figure 3D**). The non-active GA19, GA20, and GA21 showed low but steady content throughout the ripening process, from the 10 to 100% ripening stage (**Supplementary Figure S3B**).

No active cytokinin was detected in either parthenocarpic or pollinated fruit during the ripening process. However, both *cis* and *trans* Z catabolites, c-ZOG and t-ZOG, were detected during fruit ripening (**Supplementary Figure S3C**) as parthenocarpic fruit showed a maximum concentration of 98 ng/g DW t-ZOG at 8WAP, followed by low levels during later ripening stages. Pollinated fruit showed significantly higher t-ZOG content throughout the entire ripening process, from 10 to 100% ripening, with a maximum of 177 ng/g DW at the 10% ripening stage followed by a constant decline (**Supplementary Figure S3C**). c-ZOG content in both parthenocarpic and pollinated fruit declined from 25 ng/g DW and 18 ng/g DW at the 10% ripening stage to 10 ng/g DW and 7 ng/g DW at the 100% ripening stage, respectively (**Supplementary Figure S3C**).

### Hormone-Biosynthesis and Catabolism Gene Expression

**FIGURE 4:**
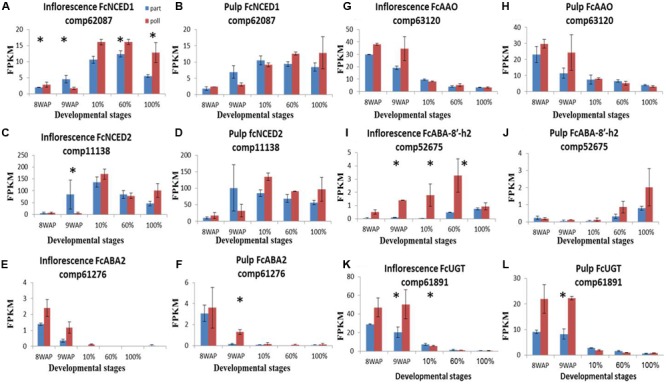
**Gene-expression pattern of six predicted ABA-metabolism genes.** Genes were identified by tblastn of the ABA-metabolism genes from *Arabidopsis thaliana* against our *Ficus carica* (Fc) data and validate against Malus and the NCBI databases. **(A–D)** Expression levels of Fc*NCED* genes in inflorescence **(A,C)** and pulp **(B,D)**. **(E,F)** Expression levels of Fc*ABA2* in inflorescence and pulp, respectively. **(G,H)** Expression levels of Fc*AAO* in inflorescence and pulp, respectively. **(I,J)** Expression levels of Fc*ABA-8′-h* in inflorescence and pulp, respectively. **(K,L)** Expression levels of Fc*UGT* in inflorescence and pulp, respectively. Bars represent standard error. Asterisk represent statistical significance determined using the edgeR test (FDR < 0.05).

To better understand the significant ripening-related concentrations of ABA (Seo and Koshiba, 2011), auxin (Mano and Nemoto, 2012) and ethylene (Owino et al., 2006; Freiman et al., 2014), we analyzed the transcript expression of each hormone-biosynthesis and catabolism gene in our fig transcriptome. The genes transcripts were tblastn vs., our database and cross validate with NCBI and Malus. Thirteen ABA-related transcripts were identified (**Supplementary Figure S4**). The ABA-synthesis *NCED, ABA2* and *AAO* transcripts showed the highest expression levels at the 10% ripening and 8WAP stages, (**Figures 4A–H**). The ABA-degradation *ABA-8′-h* gene showed later expression peaks in both pulp and inflorescence tissues at 60 and 100% ripening stages, respectively, while the alternative degradation *UGT* gene, transcript displayed a maximum expression level at 8WAP (**Figures 4I–L**). In general, differentially expressed transcripts showed higher expression levels in pollinated fruit than in parthenocarpic ones throughout the ripening process, particularly in the inflorescence (**Figure 4**). Both *NCED1* and *2* showed higher expression levels in parthenocarpic fruit inflorescence and pulp at 9WAP, before ripening onset, and higher expression levels in the pollinated fruit inflorescence at the 10% ripening stage (**Figures 4A–D**). At this stage, the *NCED1* and *2* genes were higher in the pollinated inflorescence, while *NCED1* remained higher throughout ripening (**Figures 4A–D**). *NCED2* was higher in the pollinated pulp stages at 10–100% ripening, while *NCED1* was higher in the pollinated pulp from the 60% ripening stage onward (**Figures 4B,D**). *ABA2* showed an early ripening peak at 8–9WAP with higher expression levels in pollinated vs. parthenocarpic inflorescences, followed by lower levels during the ripening stages, as shown for *UGT* and *AAO*, which were upregulated in the pollinated pulp compared to the parthenocarpic one **Figures 4E–H,K,L**).

**FIGURE 5:**
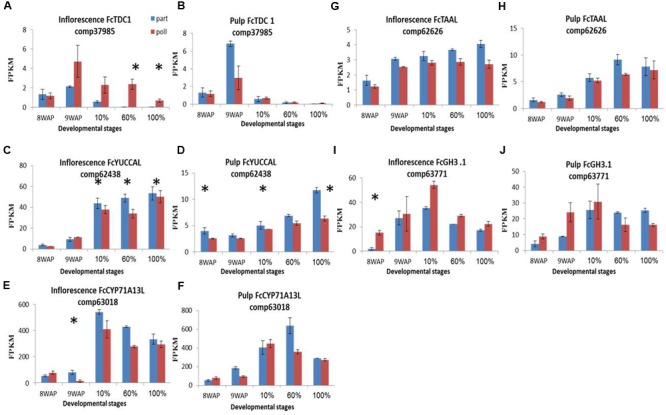
**Gene-expression pattern of five predicted IAA-metabolism genes.** Genes were idenitfied by tblastn of the IAA-biosynthesis genes from *Arabidopsis thaliana* against our *Ficus carica* (Fc) data and validate against Malus and the NCBI databases. **(A,B)** Expression levels of Fc*TDC1* in inflorescence and pulp, respectively. **(C,D)** Expression levels of Fc*YUCCAL* in inflorescence and pulp, respectively. **(E,F)** Expression levels of Fc*CYP71A13L* in inflorescence and pulp, respectively. **(G,H)** Expression levels of Fc*TAAL* in inflorescence and pulp, respectively. **(I,J)** Expression levels of Fc*GH3.1* in inflorescence and pulp, respectively. Bars represent standard error. Asterisk represent statistical significance determined using the edgeR test (FDR < 0.05).

A total of 19 auxin-biosynthesis- and catabolism-related gene transcripts were identified (**Figure 5**; **Supplementary Figures S5**). *TDC1, YUCCAL, CYP71A13, TAAL* and *GH3.1* transcripts (**Figure 5**) and IAA content showed increased expression and higher concentrations simultaneously, throughout fruit ripening (**Figure 3**). The first gene involved in IAA metabolism, *TDC1*, showed higher expression in the pollinated fruit inflorescence over the parthenocarpic ones with maximum representation 9WAP followed by a gradual decreasing throughout ripening. In the pulp *TDC1* showed higher representation at the 9WAP point in the parthenocarpic fruit over the pollinated ones (**Figures 5A,B**). *YUCCAL* expression was 10-fold higher in the inflorescence than in the pulp, with a constant increase throughout the ripening process and no significant differences between pollinated and parthenocarpic fruit. This gene, however, was upregulated in parthenocarpic pulp at both 60 and 100% ripening stages (**Figures 5C,D**). *CYP71A13L* had low expression levels in the green 8–9WAP fruit, and was generally higher in parthenocarpic than in pollinated ones. Expression levels of this gene were 10-fold higher during ripening as parthenocarpic fruit maintained their relatively higher transcript levels compared to pollinated fruit (**Figures 5E,F**). Low expression of *TAAL*, with relatively higher representation in the parthenocarpic fruit (**Figures 5G,H**), was also found when the auxin conjugation gene *GH3.1* showed a peak at the 10% ripening stage, with higher representation in the pollinated inflorescence. *GH3.1* expression was higher in pollinated pulp compared to parthenocarpic pulp during the earlier stages, while showing a significant increase at the later ripening stages in the latter (**Figures 5I,J**).

**FIGURE 6:**
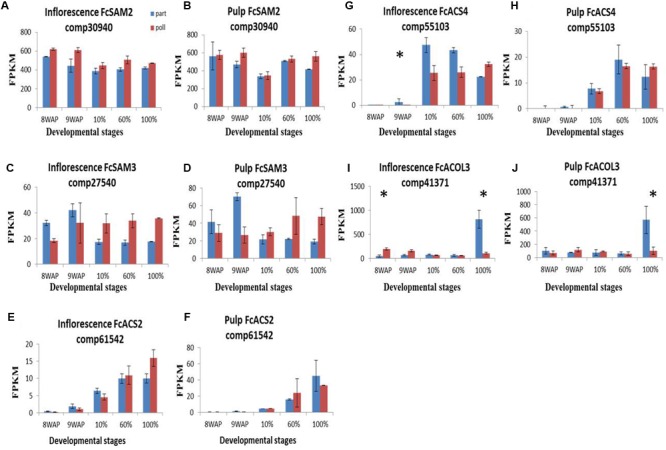
**Gene-expression pattern of six predicted ethylene-metabolism genes.** Genes were identified by tblastn of the ethylene-biosynthesis genes from *Arabidopsis thaliana* against our *Ficus carica* (Fc) data and validate against Malus and the NCBI databases. **(A–D)** Expression levels of Fc*SAM* genes in inflorescence **(A,C)** and pulp **(B,D)**. **(E–H)** Expression levels of Fc*ACS* genes in inflorescence **(E,G)** and pulp **(F,H)**. **(I,J)** Expression levels of Fc*ACOL3* in inflorescence and pulp, respectively. Bars represent standard error. Asterisk represent statistical significance determined using the edgeR test (FDR < 0.05).

Despite a previous study by Rosianski et al. (2016) showing no difference in ethylene production between parthenocarpic and pollinated fig fruit, two transcripts among the 14 ethylene-biosynthesis-related genes, SAM synthase and *SAM2* transcripts (**Figures 6A,B, Supplementary Figure S6**) showed higher expression levels in pollinated vs. parthenocarpic inflorescences during the ripening process, while *SAM3* had higher levels in parthenocarpic and pollinated fruit at 8–9WAP and 10–100% ripening stages, respectively (**Figures 6C,D**). *ACS2* and *4* showed extremely low expression levels in both pulp and inflorescence tissues at 8–9WAP and high levels at the 10–100% ripening stages. *ACS2* expression was generally higher in the pollinated inflorescence, in contrast to *ACS4* which was highly expressed in the parthenocarpic inflorescence (**Figures 6E–H**). *ACOL3* showed similar expression levels in pollinated and parthenocarpic fruit throughout fruit development with a significant expression peak at 100% ripening in parthenocarpic fruit (**Figures 6I,J**).

## Discussion

The transition of both pollinated and parthenocarpic fig fruit from the pre-ripening to ripening stage shows impressive morphological and physiological changes (Rosianskey et al., 2016). Pollinated fruit is round, larger in diameter and weight and has improved firmness as compared to the pear-shaped parthenocarpic fruit. Such changes are probably regulated by phytohormones, and are accompanied by large rearrangements of the fruit transcriptome. The transcriptome of the pollinated vs. parthenocarpic platform during fig fruit ripening provided us with a large database of 147,000 transcripts and 53,000 annotated genes. The two distinct physiological stages—green phase II and ripening phase III— were significantly different in their patterns of gene-expression, in both pulp and inflorescence tissues. Comparison of pollinated and parthenocarpic tissues during fruit ripening showed differential gene transcripts in pollinated vs. parthenocarpic fruit, particularly in the inflorescence. Twenty two and four times higher numbers of differentially represented genes in the reproductive inflorescence as compared to the vegetative pulp tissue during both phases II and III, respectively, highlight the important ripening-related role of the inflorescence. In all cases, at least twofold higher number of up-represented genes in the pollinated vs. parthenocarpic fruit was observed in the parthenocarpic vs. pollinated fruit (**Table 2**). These differences in gene expression between pollinated and parthenocarpic fruit are in agreement with the superior physiological characteristics of the bigger, heavier and tastier pollinated fruits, which also last longer in storage (Rosianski et al., 2016). Moreover, zooming in on the functions of the differently represented genes, the pollinated inflorescence and the pollinated pulp exhibit a higher number of genes that may contribute to the superior physiology of the pollinated fruit, e.g., Taste- and aroma-related genes, cell-wall storage, cell energy and chlorophyll in both phase II and phase III (**Table 2**).

Our data also show high representation of either elementary or subordinate hormone-related metabolic genes such as seed and embryo, transcription factor and cell-response genes, probably as a consequence of the pollination-induced development of rigid seeds that strengthen the pollinated inflorescence sink properties (Crane and van Overbeek, 1965; Rosianski et al., 2016) (**Table 2**). The importance of seeds’ presence in the developing fruit has been previously reported in strawberry fruit, where removal of the achenes (seeds) from unripe green fruit induced earlier fruit softening which was partially inhibited by treatment of de-achened fruit with auxin (Medina-Escobar et al., 1997; Benítez-Burraco et al., 2003).

### Differential Hormone Production in Whole Fig Fruit

**FIGURE 7:**
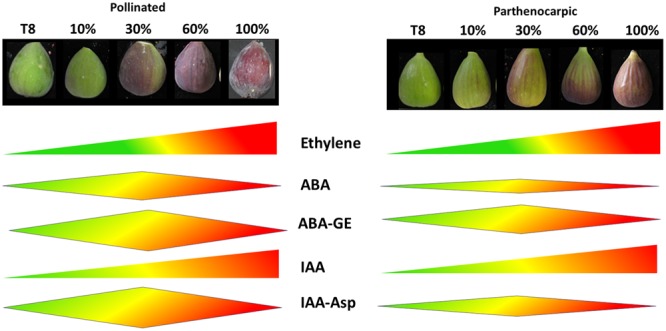
**Schematic model of hormone production during the ripening of parthenocarpic and pollinated fig fruits.** Hormone production level is expressed by schematic width. T8 represent 8 weeks after pollination, end of phase II; 10 – 10% fruit ripening; 60 – 60% fruit ripening; 100 – 100% fruit ripening.

The well-studied strong connection between seed and hormone metabolism led us to produce a comprehensive hormotome of the five common plant hormones: ethylene, ABA, auxin, GA, cytokinin and their derivatives from the onset of, and throughout ripening of the ‘common’ fig fruit cv. Brown Turkey. A schematic model of hormone production during the ripening of parthenocarpic and pollinated fruit is provided in **Figure 7**.

Ethylene production was not affected by the pollination event, as both pollinated and parthenocarpic fruit showed a typical ethylene burst at the onset of fruit ripening with no significant difference between them (Rosianski et al., 2016) (**Figure 7**). The peak of ABA production at 30% ripening (**Figures 3A** and **7**) was in agreement with its production in tomato fruit (Srivastava and Handa, 2005), where the importance of the reproductive tissue in hormone production was seen in parthenocarpic tomatoes, which had a lower proportion of ABA than their seeded counterparts (Sjut and Bangerth, 1982). Similarly, the maximum ABA concentration in the pollinated fig fruit was doubles that in parthenocarpic ones (**Figure 3A**). In addition, ABA production was higher in the pollinated fruit, expressed by higher production of its PA, DPA and ABA–GE derivatives, than in the parthenocarpic fruit (**Figures 3A** and **7, Supplementary Figure S3**).

IAA production in both pollinated and parthenocarpic ‘Brown Turkey’ initiated in phase II and remained high during the entire ripening phase III (**Figures 3B** and **7**). In the climacteric apple, IAA concentration showed a three- to fourfold increase prior to the rapid rise in ethylene concentration but fell to its original level as this rise occurred (Mousdale and Knee, 1981). The same pattern of early-growth-stage IAA production before the onset of chlorophyll degradation was reported in the non-climacteric strawberry fruit (Symons et al., 2012). In tomato, the rise and fall of IAA production also occurs prior to the ethylene peak but appears much earlier, before the onset of chlorophyll degradation at the ‘breaker’ stage when cell expansion and fruit ripening take place (Srivastava and Handa, 2005). Comparison of pollinated vs. parthenocarpic tomato fruit revealed higher production of IAA in the seeded pollinated fruit than in the parthenocarpic ones (Sjut and Bangerth, 1982). IAA production that precedes the ethylene peak at ripening was also reported by Lodhi et al. (1969) during first parthenocarpic and second pollinated crop’s phase II of ‘San-Pedro’ fig fruit, in addition to a later peak in IAA production during growth and ripening phase III. These two peaks of IAA production differ from our current results (**Figures 3B** and **7**), possibly due to the different fig cultivars or the difference in fig types—‘San-Pedro’ vs. ‘common’ type fig.

No active cytokinins or gibberellins were found in either parthenocarpic or pollinated fig fruit hormotomes. This finding is in agreement with other climacteric fresh fruit such as tomato, where GA and cytokinin contents decrease before ripening onset (Srivastava and Handa, 2005). An exception to this pattern was GA8, which was strongly induced during phase III, presumably to inhibit GA1 synthesis (**Figure 3D**).

### Tissue-Related Differential Expression of Hormone-Metabolism Genes

The higher representation of transcripts and their differential expression in the inflorescence as compared to the pulp may suggest a major role for the reproductive inflorescence tissue in controlling fruit development and ripening. In addition, the seeds in the “true” fruit contributed to the ripening, while gene expression in the vegetative pulp tissue was generally lower. This general conclusion is in agreement with previous work on ethylene control of fig fruit ripening where ethylene production was mainly controlled in the fruit inflorescence (Freiman et al., 2015). Here, two ethylene-metabolism genes were differentially represented in the fig pulp: *ACS4* and *ACOL3*. A higher representation of yet another ethylene-metabolic gene, *ACS2*, was observed in the pollinated inflorescence, in contrast to *ACS4* which was expression was higher in the parthenocarpic inflorescence, suggesting an effect of seed production on ethylene metabolism.

The ABA-production pattern during most climacteric fruits’ ripening usually precedes or parallels ethylene production (Crane and Blondeau, 1949; Lara and Vendrell, 2000; Zhang et al., 2009; Symons et al., 2012; Leng et al., 2014). For example, in the climacteric tomato fruit, ABA content increases at the cell expansion and fruit ripening and decreases before the ripening phase, which includes loss of chlorophyll color and red lycopene accumulation with no change in fruit size (Srivastava and Handa, 2005). It was shown that this peak in ABA content precedes ethylene production in tomato seeds and flesh by ∼4 days (Zhang et al., 2009; Leng et al., 2014). Similarly, the ABA peak was shown to precede ethylene production during the climacteric persimmon fruit’s ripening by 30 days, and by 10–20 days in the climacteric apple fruit (Lara and Vendrell, 2000; Leng et al., 2014). The unique fig fruit exhibited similar characteristics, with elevated ABA production at the beginning of the ripening period in parthenocarpic and pollinated fruit (**Figure 3A**). ABA production paralleled the onset of ethylene production which peaked 1 day later, at 30% ripening. In contrast, climacteric fig fruit also possess characteristics that can be related to non-climacteric fruit (Sozzi et al., 2005; Owino et al., 2006; Freiman et al., 2014). In our study, the increase in ABA content is not only seems to activate the ripening process as in climacteric fruit, but also coincided with increased fruit size as in non-climacteric strawberry (Symons et al., 2012). The increase in ABA content started at the end of the quiescence phase II throughout maturation phase III (fruit size 40–55 mm). The advanced ABA production in the pollinated vs. parthenocarpic fruit was reflected in the representations of ABA-metabolism genes, including the key gene in ABA production, *NCED* (**Figures 3A** and **4**). The importance of the fig inflorescence *NCED1* and *2* transcripts, which showed up-representation before *ACS2* and *4*, is in agreement with tomato fruit, where ABA induces ethylene biosynthesis via the regulation of *ACS* and *ACO* expression (Zhang et al., 2009).

The pattern of *TDC1, CYP73A13* and *TAAL* auxin-metabolism gene transcripts in the fig transcriptome was in agreement with the profile of auxin content in the fig hormotome, which increased during fruit ripening with no general difference for pollinated vs. parthenocarpic fruit (**Figures 3, 5** and **7**). In contrast, higher representation in the pollinated fruit inflorescence compared to its parthenocarpic counterpart was seen for *GH3.1*, the gene responsible for IAA–Asp conjugation (**Figures 5I,J**). The non-active IAA–Asp conjugate, suggested to directly affect responses to abiotic stress, was found at higher levels in the pollinated vs. parthenocarpic fruit.

## Conclusion

In this study, we characterized the physicochemical features and transcriptional profiles associated with the development of ripening capacity in fig before and across ripening stages. Our analysis, which focused mainly on the differential expression in two tissues, the inflorescence and the fruit pulp, of genes associated with hormone signaling, suggested a role for specific transcripts that are mainly represented in the fruit inflorescence. This suggests that the reproductive part of the fig syconium is the main coordinator of fruit ripening. Surprisingly, there was much less differences in gene expression between parthenocarpic and pollinated fruit in the pulp, suggesting that the reproductive component of the fig syconium is the main coordinator of fruit ripening, and that seed development in the pollinated fruit controls these differences mainly via hormonal coordination. Since ABA seems to be the upstream operator of the processes of fig fruit ripening, we plan to further investigate its role in additional experimental systems and genotypes.

## Author Contributions

YR and MF designed the experiments. YR, YD, and ZF conducted the experiments. YR, ZK and AD-F interpreted the results. MF and SM-C, YR, KL, and ZF prepared the manuscript. All authors have read and approved the manuscript for publication.

## Conflict of Interest Statement

The authors declare that the research was conducted in the absence of any commercial or financial relationships that could be construed as a potential conflict of interest.

## Footnotes

1 http://www.nrc-cnrc.gc.ca

2 http://hannonlab.cshl.edu/fastx_toolkit/index.html

3 http://www.geneontology.org

## Supplementary Material

FIGURE S1 **Biological process, cellular components and molecular functions distribution in the transcriptome as analyzed by the Blast2GO tool through the Combined Graph Display. (A–C)** distributions of biological process, cellular components and molecular functions in high level of GO terms. **(D–F)** distributions of biological process, cellular components and molecular functions in lower level of GO terms.

FIGURE S2 **Hierarchically clustered significantly differentially expressed transcripts after grouping into four clusters.** Expression patterns for the transcripts in each cluster (log2-transformed, median-centered, average of two replications). Each transcript is plotted (gray) along with the mean expression profile for that cluster (blue). **(A)** Parthenocarpic inflorescence. **(B)** Pollinated inflorescence. **(C)** Parthenocarpic pulp. **(D)** Pollinated pulp.

FIGURE S3 **Hormone derivative production during parthenocarpic and pollinated fig fruit development. (A)** ABA degradation derivative 7′OH-ABA, PA and DPA. **(B)** Non-active gibberellins GA19, GA20 and GA29. **(C)** Non-active cytokinin derivative (*trans*) t-ZOG and (*cis*) c-ZOG. Average of three biological repeats of 15 fruits per treatment ± SE. part, parthenocarpic; poll, pollinated. Bars represent standard error. Asterisk represent statistical significance determined using Student’s *t*-test.

FIGURE S4 **Heat map of the log2-normalized transcriptomic representation profiles of 13 ABA metabolism-related gene transcripts in parthenocarpic and pollinated fruit inflorescence and pulp.** Red, up-representation; green, down-representation. 8WAP–9WAP, 8–9 weeks after pollination; 10, 60, 100%, ripening stage.

FIGURE S5 **Heat map of the log2-normalized transcriptomic representation profiles of 19 auxin metabolism-related gene transcripts in parthenocarpic and pollinated fruit inflorescence and pulp.** Red, up-representation; green, down-representation. 8WAP–9WAP, 8–9 weeks after pollination; 10, 60, 100%, ripening stage.

FIGURE S6 **Heat map of the log2-normalized transcriptomic representation profiles of 13 ethylene metabolism-related gene transcripts in parthenocarpic and pollinated fruit inflorescence and pulp.** Red, up-representation; green, down-representation. 8WAP–9WAP, 8–9 weeks after pollination; 10, 60, 100%, ripening stage.

TABLE S1 **Number of down- and upregulated enriched molecular functions in pollinated and parthenocarpic fig inflorescence and pulp**.
