# Supplementary material for: Tissue-Specific Transcriptome and Hormonal Regulation of Pollinated and Parthenocarpic Fig (Ficus carica L.) Fruit Suggest that Fruit Ripening Is Coordinated by the Reproductive Part of the Syconium
Source: Front Plant Sci. 2016 Nov 29;7:1696. doi: 10.3389/fpls.2016.01696 (PMC5126050; doi:10.3389/fpls.2016.01696)
Supplement: FIGURE S1 — Biological process, cellular components and molecular functions distribution in the transcriptome as analyzed by the Blast2GO tool through the Combined Graph Display. (A–C) distributions of biological process, cellular components and molecular functions in high level of GO terms. (D–F) distributions of biological process, cellular components and molecular functions in lower level of GO terms. [file Data_Sheet_1.zip › Image 1.PDF]

Supplementary Figure 1.

A

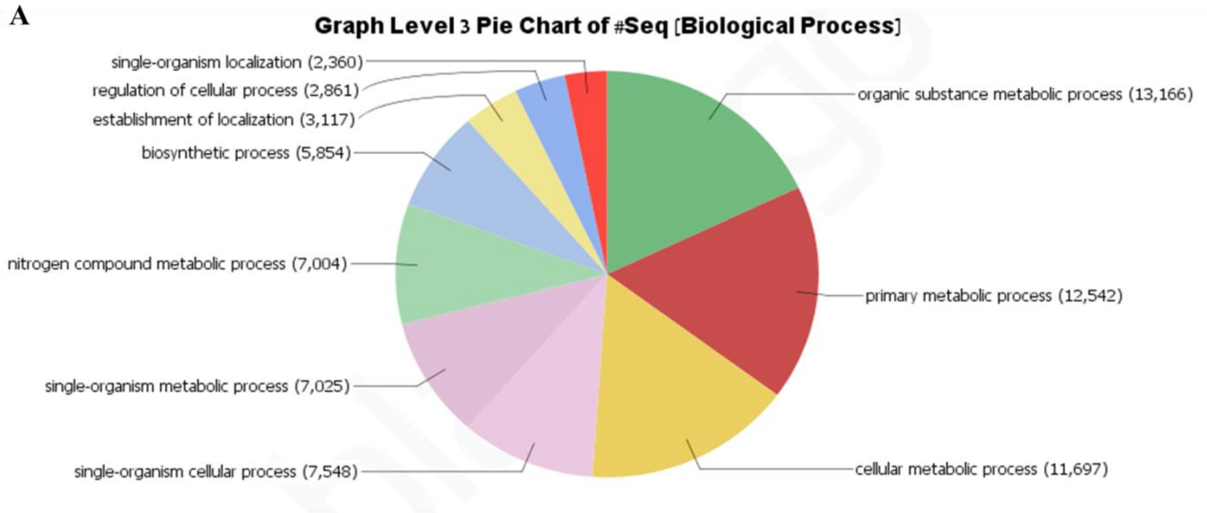

B

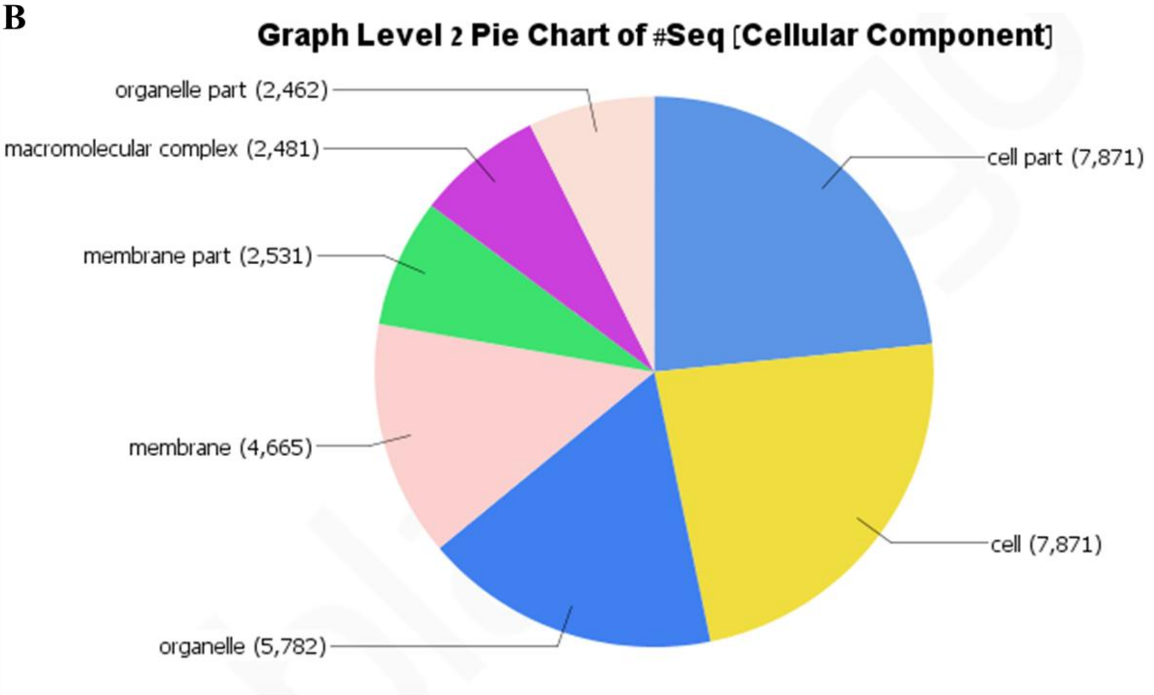
Graph Level 2 Pie Chart of #Seq [Cellular Component]
organelle part (2,462)
macromolecular complex (2,481)
membrane part (2,531)
membrane (4,665)
organelle (5,782)
cell part (7,871)
cell (7,871)

C

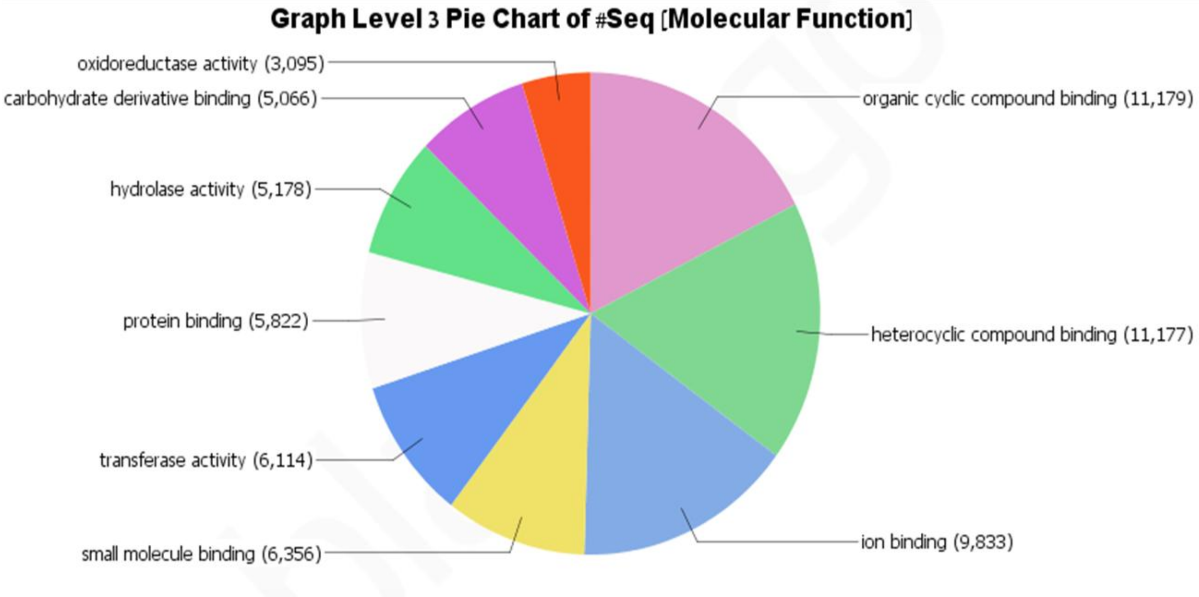
Graph Level 3 Pie Chart of #Seq [Molecular Function]
oxidoreductase activity (3,095)
carbohydrate derivative binding (5,066)
hydrolase activity (5,178)
protein binding (5,822)
transferase activity (6,114)
small molecule binding (6,356)
organic cyclic compound binding (11,179)
heterocyclic compound binding (11,177)
ion binding (9,833)

D

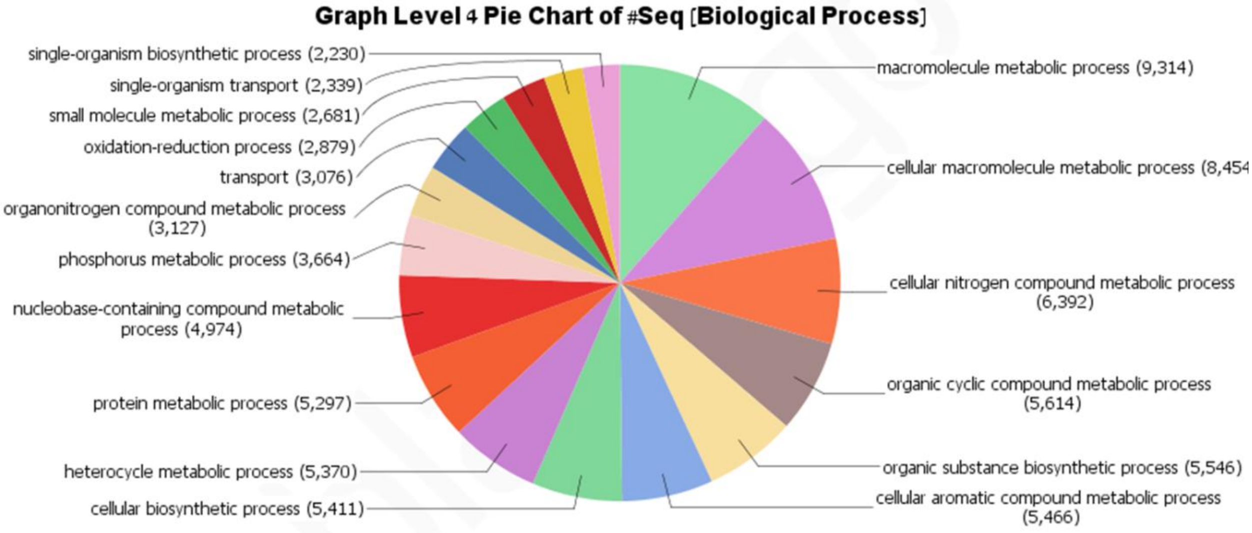
Graph Level 4 Pie Chart of #Seq [Biological Process]
single-organism biosynthetic process (2,230)
single-organism transport (2,339)
small molecule metabolic process (2,681)
oxidation-reduction process (2,879)
transport (3,076)
organonitrogen compound metabolic process (3,127)
phosphorus metabolic process (3,664)
nucleobase-containing compound metabolic process (4,974)
protein metabolic process (5,297)
heterocycle metabolic process (5,370)
cellular biosynthetic process (5,411)
macromolecule metabolic process (9,314)
cellular macromolecule metabolic process (8,454
cellular nitrogen compound metabolic process (6,392)
organic cyclic compound metabolic process (5,614)
organic substance biosynthetic process (5,546)
cellular aromatic compound metabolic process (5,466)

E

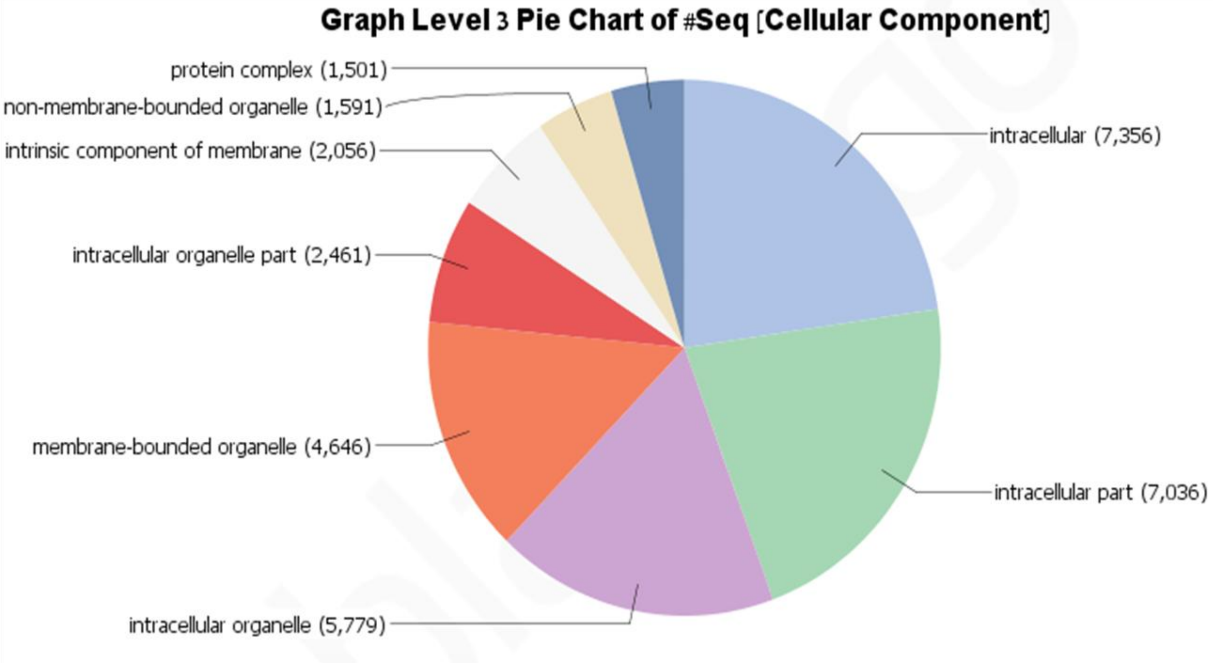
Graph Level 3 Pie Chart of #Seq [Cellular Component]
protein complex (1,501)
non-membrane-bounded organelle (1,591)
intrinsic component of membrane (2,056)
intracellular organelle part (2,461)
membrane-bounded organelle (4,646)
intracellular organelle (5,779)
intracellular (7,356)
intracellular part (7,036)

F

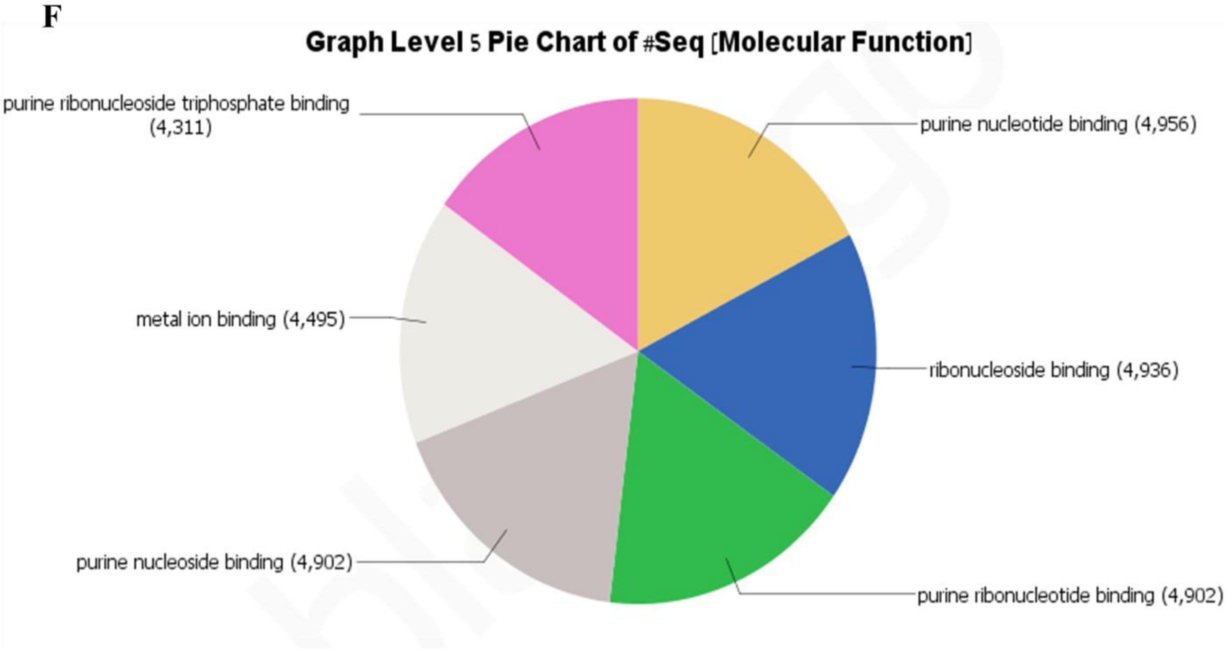
Graph Level 5 Pie Chart of #Seq [Molecular Function]
purine ribonucleoside triphosphate binding (4,311)
purine nucleotide binding (4,956)
metal ion binding (4,495)
ribonucleoside binding (4,936)
purine nucleoside binding (4,902)
purine ribonucleotide binding (4,902)
