# Supplementary material for: Tissue-Specific Transcriptome and Hormonal Regulation of Pollinated and Parthenocarpic Fig (Ficus carica L.) Fruit Suggest that Fruit Ripening Is Coordinated by the Reproductive Part of the Syconium
Source: Front Plant Sci. 2016 Nov 29;7:1696. doi: 10.3389/fpls.2016.01696 (PMC5126050; doi:10.3389/fpls.2016.01696)
Supplement: FIGURE S1 — Biological process, cellular components and molecular functions distribution in the transcriptome as analyzed by the Blast2GO tool through the Combined Graph Display. (A–C) distributions of biological process, cellular components and molecular functions in high level of GO terms. (D–F) distributions of biological process, cellular components and molecular functions in lower level of GO terms. [file Data_Sheet_1.zip › Table 1.pdf]

Supplementary Table 1.

	poll fl up	part fl up	poll fl down	part fl down	poll pulp up	part pulp up	poll pulp down	part pulp down		
**cell wall**	2	3	8	2	3	2	2	2		
**oxidation**	3	4	1	1	7	5	3	2		
**protein metabolism**	12	18	1	6	22	17	8			
**cellolar response**	3	20	5	6	33	20	6			
**hormone**	1	4			7	5				
**energy**	2	6			3	1				
**secondary and color**		1	2	2	1	2				
**fertilization**			2							
**lipid**	1		3		2					
**total**	24	56	22	17	68	52	19	4	total part	total poll
			46	73			87	56	133	129
