# Supplementary figures and images for: Tissue-Specific Transcriptome and Hormonal Regulation of Pollinated and Parthenocarpic Fig (Ficus carica L.) Fruit Suggest that Fruit Ripening Is Coordinated by the Reproductive Part of the Syconium
Source: Front Plant Sci. 2016 Nov 29;7:1696. doi: 10.3389/fpls.2016.01696 (PMC5126050; doi:10.3389/fpls.2016.01696)

Supplementary Figure 2.

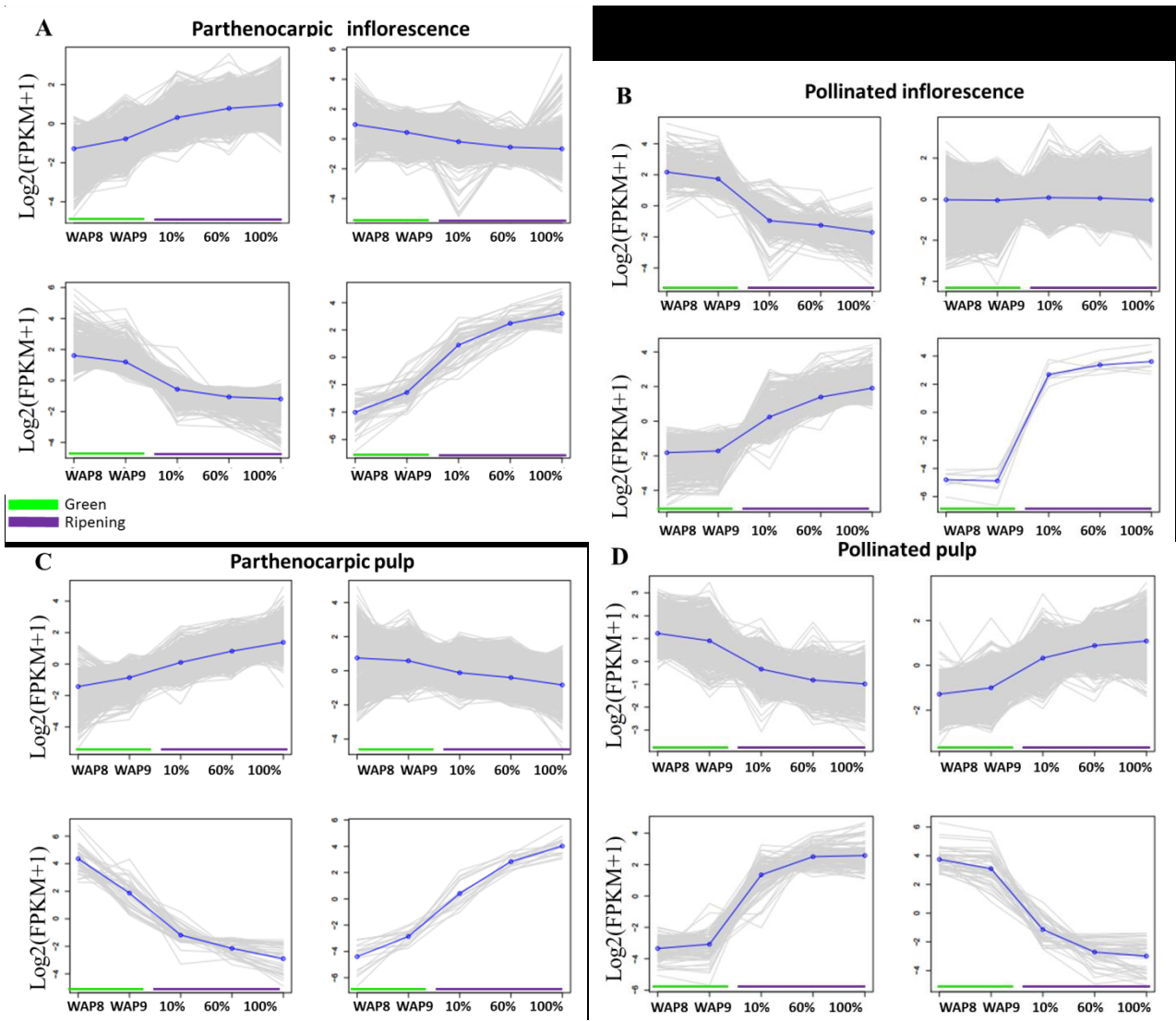

Supplement: FIGURE S1 — Biological process, cellular components and molecular functions distribution in the transcriptome as analyzed by the Blast2GO tool through the Combined Graph Display. (A–C) distributions of biological process, cellular components and molecular functions in high level of GO terms. (D–F) distributions of biological process, cellular components and molecular functions in lower level of GO terms. [file Data_Sheet_1.zip › Image 2.PDF]

Supplementary Figure 3.

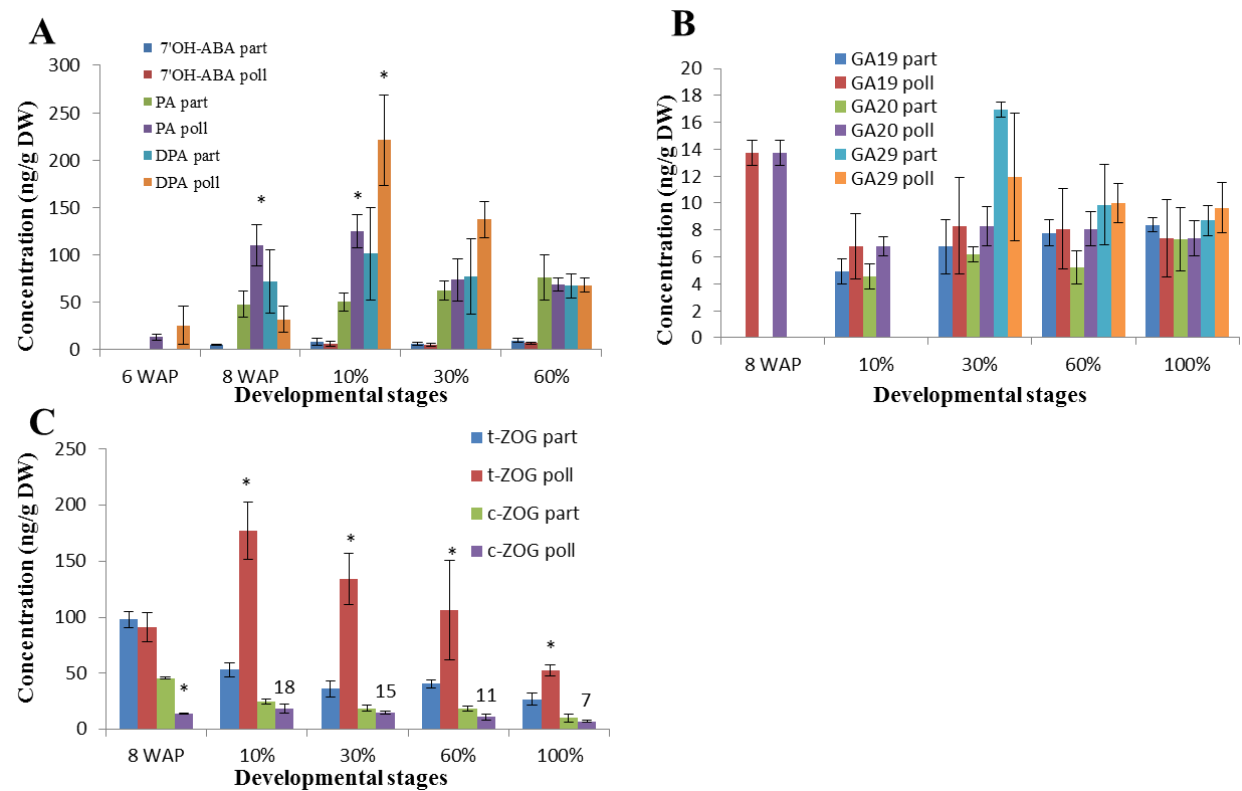

Supplement: FIGURE S1 — Biological process, cellular components and molecular functions distribution in the transcriptome as analyzed by the Blast2GO tool through the Combined Graph Display. (A–C) distributions of biological process, cellular components and molecular functions in high level of GO terms. (D–F) distributions of biological process, cellular components and molecular functions in lower level of GO terms. [file Data_Sheet_1.zip › Image 3.pdf]

Supplementary Figure 4.

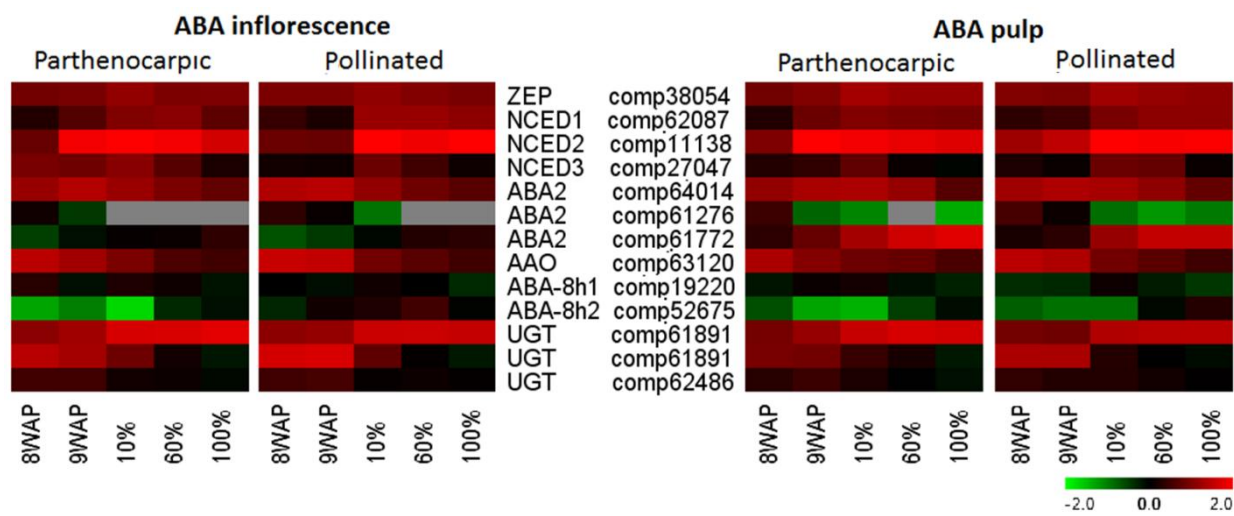

Supplement: FIGURE S1 — Biological process, cellular components and molecular functions distribution in the transcriptome as analyzed by the Blast2GO tool through the Combined Graph Display. (A–C) distributions of biological process, cellular components and molecular functions in high level of GO terms. (D–F) distributions of biological process, cellular components and molecular functions in lower level of GO terms. [file Data_Sheet_1.zip › Image 4.pdf]

Supplementary Figure 5.

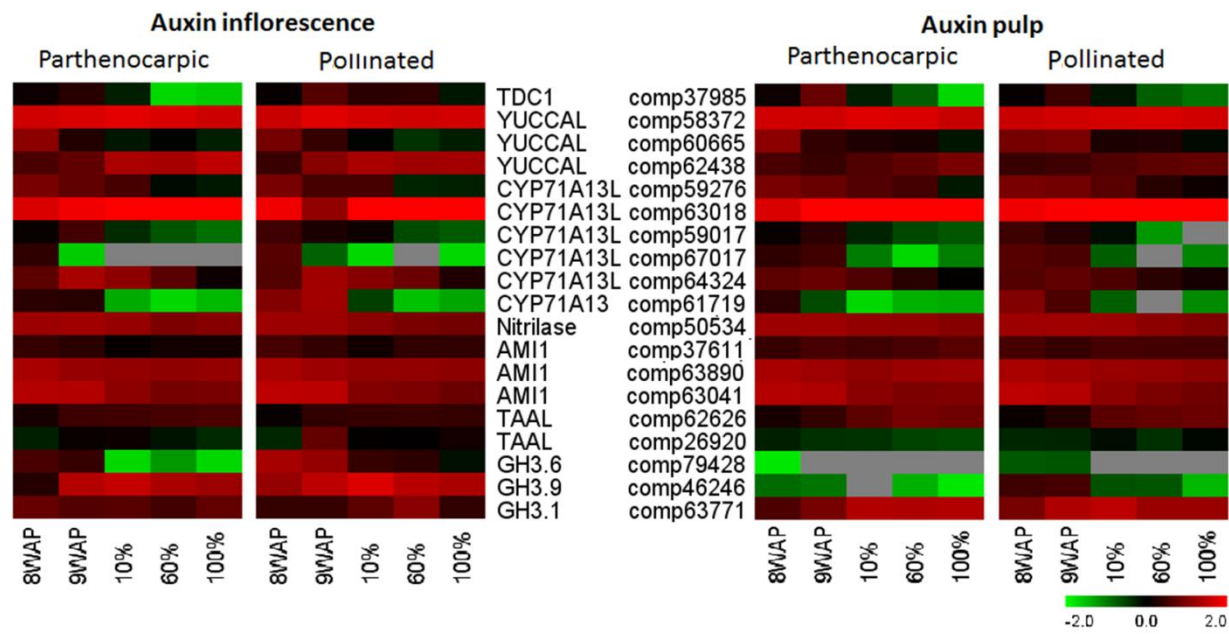

Supplement: FIGURE S1 — Biological process, cellular components and molecular functions distribution in the transcriptome as analyzed by the Blast2GO tool through the Combined Graph Display. (A–C) distributions of biological process, cellular components and molecular functions in high level of GO terms. (D–F) distributions of biological process, cellular components and molecular functions in lower level of GO terms. [file Data_Sheet_1.zip › Image 5.pdf]

Supplementary Figure 6.

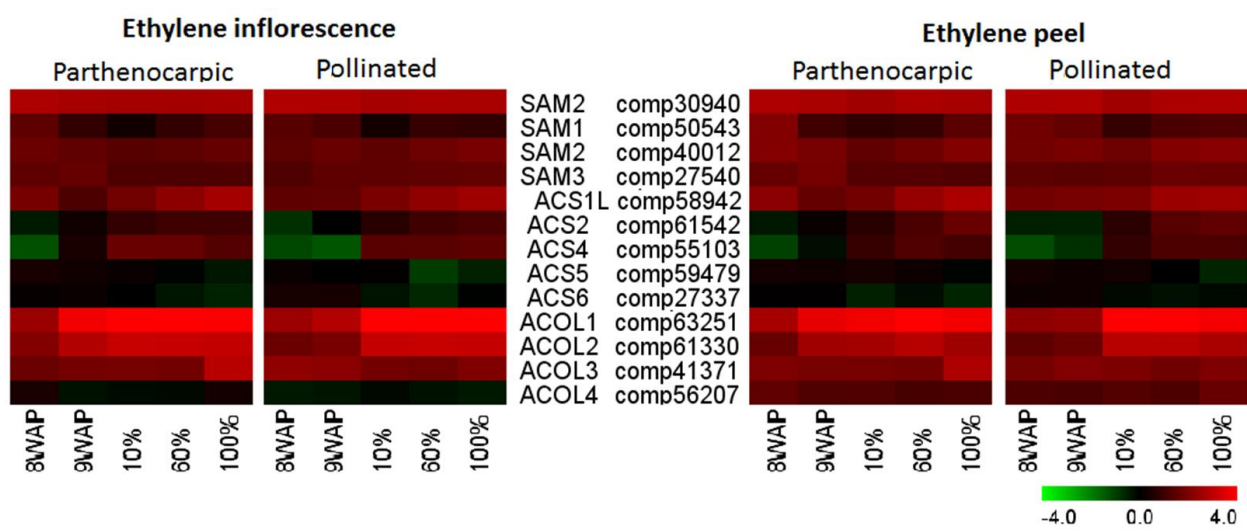

Supplement: FIGURE S1 — Biological process, cellular components and molecular functions distribution in the transcriptome as analyzed by the Blast2GO tool through the Combined Graph Display. (A–C) distributions of biological process, cellular components and molecular functions in high level of GO terms. (D–F) distributions of biological process, cellular components and molecular functions in lower level of GO terms. [file Data_Sheet_1.zip › Image 6.pdf]
